# “Not as Bad as I Thought”: Undergraduate Students’ Perspectives on Aging

**DOI:** 10.1093/geroni/igaa057.015

**Published:** 2020-12-16

**Authors:** Nasreen Sadeq

**Affiliations:** University of South Florida, Tampa, Florida, United States

## Abstract

Ageism remains prevalent in our society and negatively affects older adults. Undergraduate education could be a potential avenue for combating ageism among individuals moving into the workforce. The current investigation is a pilot study in which students enrolled in an upper-level undergraduate course were asked to reflect on how their perspectives on aging had changed following the completion of the course. The course, Psychology of Aging, provides students with an overview of the psychological aspects of aging, including cognitive, physical, and social changes. Consensual qualitative methods were used to explore student responses (N=150) and analyzed using Atlas.ti 7. The results indicated that majority of students had a more positive view of aging following course completion. The three most common themes that emerged were 1) positive aging, 2) living arrangements, and 3) social relationships. For the positive aging theme, students recognized the positive aspects of aging, with many commenting that aging was not as bad as they originally thought. Nearly all the responses in the living arrangements theme reflected students’ previous assumptions that most older adults live in nursing homes. In the social relationships theme, students realized that aging does not make people value social connection any less. The findings suggest that providing students with accurate information about aging has the potential to correct their negative views of aging, and highlight the importance of gerontology coursework in higher education, particularly for those pursuing careers in which they will regularly interact with older adults.

